# Inhibition by ATP regulates the activity of a CBASS anti-phage nucleotide cyclase

**DOI:** 10.1042/BCJ20260457

**Published:** 2026-07-30

**Authors:** Laura Gaskell-Mew, Stuart McQuarrie, Stephen A. McMahon, Peter Wotherspoon, Shirley Graham, Tracey M. Gloster, Malcolm F. White

**Affiliations:** School of Biology, University of St Andrews, St Andrews KY16 9ST, U.K.

**Keywords:** adenylate cyclase, bacteriophages, immune response, nucleic acids

## Abstract

The bacterial anti-phage immune system is complex, diverse, and in several important cases ancestral to that found in eukaryotes, including humans. One example is CBASS (cyclic oligonucleotide based anti-phage signalling system), a widespread bacterial defence that signals phage presence in the cell via cyclic nucleotide second messengers, activating ancillary effectors to combat infection. CBASS is homologous and ancestral to the eukaryotic cGAS/STING pathway for antiviral defence. The heart of the system is a nucleotide cyclase known as a cGAS/DncV-like nucleotidyltransferase, which is activated by phage infection. The mechanisms of activation of CBASS cyclases are diverse and in most cases not fully understood at a molecular level. Moreover, it is vital to keep these signal-generating enzymes fully inactive in the absence of phage infection to avoid auto-toxicity. Here, we report a structural and mechanistic study of a CBASS cyclase from *Bacillus cereus*. Using crystal structures of key reaction intermediates, coupled with kinetic analyses, we show that the substrate, ATP, plays a fundamental role in the inhibition of the non-activated form of the enzyme *in vitro*. We provide a molecular explanation for this regulation and explore the implications for the regulation of these important defence systems in bacterial immunity.

## Introduction

Recent years have witnessed a revolution in our understanding of the bacterial immune system, with over 150 different defence systems discovered [1–3]. The average bacterium encodes over six defence systems, the most common of which are restriction-modification, CRISPR and CBASS (cyclic oligonucleotide based antiphage signalling systems) [2,4–6]. The heart of CBASS defence is a nucleotide cyclase of the polymerase-β superfamily. The founding member is the DncV (dinucleotide cyclase in *Vibrio*) protein, which is related to the metazoan innate immunity protein cGAS [7], and synthesises 3ʹ3ʹ-cGAMP [8] using its catalytic SMODS (second messenger oligonucleotide or dinucleotide synthetase) domain [9]. The family of prokaryotic cGAS/DncV-like nucleotidyltransferases (CD-NTases) can synthesise a wide range of cyclic nucleotide (cNT) products, using all four nucleotide triphosphates, utilising both 3ʹ-5ʹ and 2ʹ-5ʹ linkages and different ring sizes [10,11]. Taking these variables into account, there is the potential to generate >180 distinct cNT species, primarily for anti-viral defence [11,12]. This diversity may have been driven by the pressure exerted by phage anti-defence proteins that degrade or bind (‘sponge’) the cNT molecules [13–16].

Augmenting this are a broad range of effector proteins, which use a sensor domain to bind specific cNTs, coupled with an effector domain that elicits a downstream response (reviewed in [17,18]). Effector domains are typically non-specific, degradative enzymes that target important cellular constituents including DNA, RNA, lipids, and nucleotide cofactors. Sequence analysis allowed the definition of eight CD-NTase clades (A–H), and biochemical screening of representative members of these clades identified the cNT products associated with each [11]. All CD-NTase family members catalyse metal-dependent phosphodiester bond formation between two nucleotide species bound in adjacent donor and acceptor binding pockets [11,19–23]. Select members of the CD-NTase family have been structurally and mechanistically characterised in detail, allowing the definition of specific active site sequence motifs that correspond to major cNT reaction products [24].

The activation of CBASS CD-NTases in response to phage infection typically results in licensing of highly toxic, non-specific effectors that degrade or disrupt cellular components, including DNA, RNA, key biomolecules, and membrane integrity (reviewed in [17,25]). This response typically leads to growth arrest, dormancy, or cell death, which prevents the completion of phage replication cycles in a process known as abortive infection. The mechanism of cyclase regulation varies widely between different CBASS systems. Examples include binding of specific phage proteins [26], metal ion concentration [27], targeted proteolysis [28], phage structural RNAs [22,29], folates [30,31], and protein–protein conjugation [30,31].

**Figure 1:**
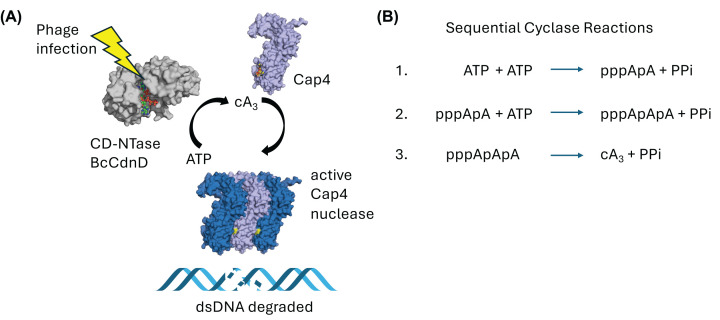
*Bacillus cereus* CBASS antiphage defence (**A**) An unknown signal of phage infection activates the CD-NTase, which generates the cA_3_ second messenger, which binds and activates Cap4 to provide immunity by degrading dsDNA. The Cap2/3 conjugation machinery is not shown. (**B**) Sequential reactions catalysed by the CD-NTase to generate cA_3_.

Recently, we described a type II CBASS system from *Bacillus cereus* that uses the Cap2 and Cap3 accessory proteins [31] to conjugate/deconjugate the CD-NTase and phage shock protein A [30]. The CD-NTase was shown to generate a 3ʹ3ʹ3ʹ- cA_3_ product, activating the Cap4 effector, which has a cA_3_-binding SMODS-associated and fused to various effector domains domain and a C-terminal nuclease domain [32]. Self-association on cA_3_ binding activates the Cap4 nuclease activity, which degrades dsDNA to provide defence (Figure 1A). Activation of the cyclase results in a series of sequential phosphotransferase reaction steps that ultimately generate cA_3_ (Figure 1B). The phage signal and molecular processes underlying activation of the CD-NTase are not understood. Here, we focus on the structure and mechanism of the *B. cereus* CD-NTase, revealing new details concerning the reaction mechanism and the observation of substrate inhibition by ATP, with implications for antiphage defence *in vivo*.

## Results

### Crystal structures of *B. cereus* CD-NTase in complex with ATP and pppApA

We expressed and purified the wild-type and inactive D73N and D75N variants of *B. cereus* CD-NTase (BcCdnD) for structural and biochemical analysis (Supplementary Figure S1). Nucleotide cyclases of the CD-NTase family bind two nucleotide triphosphates in adjacent donor and acceptor sites, catalysing the formation of a phosphodiester bond between the two molecules, with the release of pyrophosphate. Some CD-NTases generate cyclic dinucleotides, while other family members add a third nucleotide to the linear chain before cyclising the molecule. In both cases, cyclisation is a sequential reaction that requires a reorientation of the linear intermediate(s) in the active site to position the 2ʹ or 3ʹ hydroxyl of the acceptor adjacent to the alpha phosphate of the donor. The donor site appears well conserved in crystal structures of several CD-NTases, however the nucleotide in the acceptor site is correctly positioned in some structures [11,23,33] but rotated into a non-productive conformation in others [24]. Previously, we showed that the *B. cereus* cyclase generates 3ʹ3ʹ3ʹ cA_3_, which activates the cognate Cap4 effector as well as the CRISPR-associated NucC effector [30].

**Figure 2:**
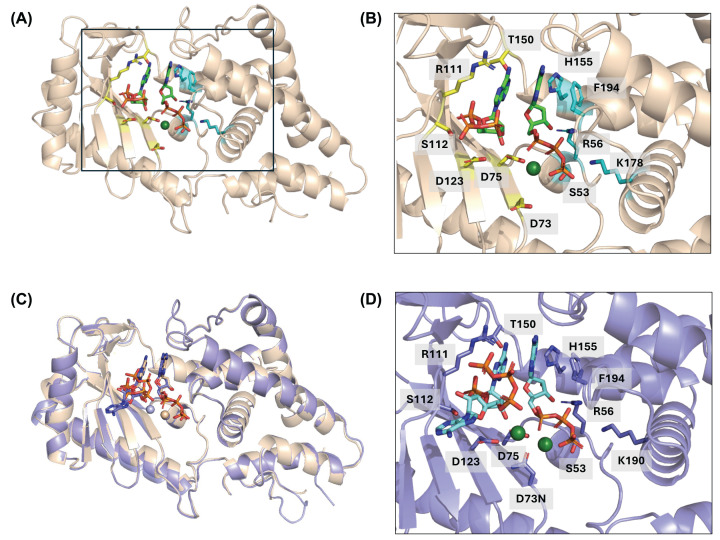
Crystal structure of BcCdnD in complex with different nucleotides (**A**) Structure of WT BcCdnD co-crystallised with ATP, showing an ATP molecule in both the donor and acceptor sites. The protein is shown in a cartoon (wheat). Conserved residues implicated in binding and catalysis are shown as sticks, coloured by cyan for donor site residues and yellow for acceptor site residues. ATP molecules are shown as sticks in green, and the magnesium ion is shown as a dark green sphere. (**B**) Closer view of the two ATP molecules binding to BcCdnD, representing the boxed-in panel (A), with the same colouring and residues labelled. (**C**) Superposition of WT BcCdnD in complex with two ATP molecules (wheat) and BcCdnD D73N variant in complex with ATP in the donor site and pppApA in the acceptor site (light blue) (root mean square deviation (RMSD) of 0.4 Å over 304 Cα atoms). Ligands and magnesium ions are shown in the same colouring as the protein backbone. (**D**) Structure of BcCdnD (D73N variant) co-crystallised with ATP in the donor site and pppApA in the acceptor site. The protein is shown in a cartoon (light blue). Conserved residues implicated in binding and catalysis are shown as sticks and labelled. ATP and pppApA are shown as sticks in cyan, and the two magnesium ions are shown as dark green spheres.

Sequence alignments (Supplementary Figure S2) suggest that the *B. cereus* CD-NTase has similarities to both clade C and D enzymes but has higher overall sequence identity (∼30%) with clade D enzymes, and hereafter will be called BcCdnD. To help elucidate the catalytic mechanism of BcCdnD, we co-crystallised the wild-type protein with ATP and the catalytically inactive D73N variant with the pppApA reaction intermediate, or both ATP and pppApA. The structure resulting from wild-type BcCdnD co-crystallised with ATP showed two molecules of ATP bound in the active site, with a molecule in each of the donor and acceptor sites (Figure 2A,B). The lack of turnover by the enzymes suggests that the crystallisation conditions may have precluded catalysis. The two molecules are positioned for 3ʹ-5ʹ phosphodiester bond formation with the acceptor ATP in the productive conformation, as seen in *Vibrio cholerae* DncV [33]. In contrast, some CD-NTases crystallise with the acceptor ATP in a non-productive conformation, for reasons that are not clear [24]. A single magnesium ion coordinates D75 and each of the phosphate groups in the ATP molecule in the donor site. The helical lid sitting above the nucleobases has conserved residues T150 and H155 at the Lid1 and Lid2 positions, respectively, as defined by Govande et al. [24] (Supplementary Figure S2). F194 stacks with the adenosine ring of the ATP molecule in the donor site, equivalent to Y250 in *Enterobacter cloacae* CdnD [24]. The adenosine moieties in both ATP molecules also stack with each other. Conserved residues S53, R56, H155 and K178 contribute to the binding pocket for ATP in the donor position, while the second ATP molecule interacts with R111, S112, D123, T150 and D75 in the acceptor site (Figure 2B and Supplementary Figure S3A). Previous studies have highlighted a large increase in enzyme thermal stability when a nucleotide is bound in the donor site of CD-NTases, but a lesser effect for the acceptor site [24]. This observation is consistent with the structural data. The B-factor, which reports on the positional flexibility of each atom in a molecule, generally correlates with binding affinity. A molecule with higher affinity binding implies stronger interactions with residues in the binding site and therefore less movement of its atoms and a lower B-factor. The average B-factor (across all atoms of each molecule in asymmetric unit) of the ATP in the donor site of BcCdnD is ∼30% lower compared with the ATP in the acceptor site and ∼20% lower compared with pppApA in the acceptor site. The higher binding affinity of ATP in the donor site could arise from the magnesium ion that is consistently observed to interact with its phosphate groups.

The overall structure of BcCdnD D73N in a complex with ATP + pppApA is essentially identical to the structure with the two ATP molecules, with an RMSD of 0.4 Å over 304 Cα atoms (Figure 2C). The initial reaction catalysed by BcCdnD generates a pppApA intermediate bridging the donor and acceptor pockets, which must disengage from the active site and rebind in the acceptor site, along with a new ATP molecule in the donor site before the second reaction can proceed. Electron density revealed binding of ATP in the donor site and pppApA in the acceptor site (in three of four molecules in the asymmetric unit; there was no ATP in the donor site and only a partial pppApA could be modelled in the acceptor site for the other). The pppApA moiety is poised for reaction with ATP and formation of the second phosphodiester bond (Figure 1B).

Interactions made between ATP + pppApA and BcCdnD are essentially identical to those observed with the two ATP molecules (Figure 2D and Supplementary Figure S3B). No additional interactions are formed by pppApA compared with ATP, which is consistent with both being tolerated as acceptor molecules. The CD-NTase family of enzymes utilizes a two-metal ion mechanism for nucleotide coupling (reviewed in [34]). The structure with ATP + pppApA shows two magnesium ions bound in the active site, as opposed to one in the structure with the two molecules of ATP. One of these is in the same position in both structures, interacting with D75 and the three phosphate groups in the ATP bound in the donor site to shield the negative charge. Additionally, this magnesium ion coordinates an asparagine, which is present following the mutation of BcCdnD to the inactive D73N variant. The structure with two ATP molecules bound was obtained with wild-type protein, and D73 points in the opposite direction, meaning an equivalent interaction is not observed. The second magnesium ion coordinates D73N, D75, D123, the α-phosphate of the ATP in the donor site and the C3ʹ-OH group of the ribose in pppApA situated in the acceptor site.

We also succeeded in determining the structure of BcCdnD (D73N variant) bound to pppApA, which superimposed with the BcCdnD structure with ATP + pppApA with an RMSD of 0.7 Å over 296 Cα atoms (Supplementary Figure S4A). Interestingly, we did not observe pppApA bound across both the donor and acceptor sites, which would be the product of conjugation of two ATP molecules, suggesting binding in that orientation is less favoured, as expected for a reaction product. Rather, pppApA was bound in the acceptor site, adopting a very similar position and interactions to that observed in the BcCdnD structure with ATP and pppApA (Supplementary Figures S3C and S4B). The terminal phosphate groups do not interact directly with the enzyme and therefore are more flexible.

### Comparison of BcCdnD with other CD-NTases

A search for the closest structural homologues of BcCdnD revealed matches with the CD-NTases from *Enterobacter cloacae* [24,35] (hereafter EcCdnD; PDB 7D4U; RMSD of 1.7 Å over 281 Cα atoms) [35] and *Salmonella enterica* (hereafter SeCdnD; PDB 7LJM; RMSD of 1.7 Å over 286 Cα atoms) [24] (Supplementary Figure S5A). The top hits are classified as clade D CD-NTases, which reaffirms the proposed classification of the *B. cereus* enzyme in clade D, as predicted from sequence alignments (Supplementary Figure S2). The nucleotides bound to each of the structures (ATP + pppApA for BcCdnD, two ATP molecules for EcCdnD, and two molecules of GTP for SeCdnD) overlap closely (Supplementary Figure S5B).

The structural comparisons give useful insights into features that govern the clade classification and substrate specificity. Govande et al. proposed key sequence features and structural motifs of CD-NTases, which predict nucleotide substrate specificity [24]. For BcCdnD, the active site motifs ‘PDVDI’ (P72–P76) and ‘LDL’ (although MDL in BcCdnD; M122–L124) are consistent with other clade D enzymes. The ‘XGSX’ motif and lid residues, however, are more predictive of substrate specificity. The ‘XGSX’ motif is SGSY (S51–Y54) in BcCdnD, similar to the TGSY sequence in *Pseudomonas aeruginosa* CdnD [36] (PaCdnD; PDB 6P8J; RMSD of 2.8 Å over 266 Cα atoms with BcCdnD) and SGSL sequence in CdnC from *Escherichia coli* [36] (EcCdnC; PDB 6P80; RMSD of 2.5 Å over 255 Cα atoms with BcCdnD), which both also generate a cA_3_ product [24]. Typically, family D enzymes such as EcCdnD and SeCdnD, which synthesise cAAG as the major product, have a QGSI motif at the equivalent position (Supplementary Figures S2 and S6A). The asparagine in these enzymes forms either one hydrogen bond with the adenine base (of ATP) or two hydrogen bonds with guanine (of GTP) in the acceptor subsite, while serine or threonine are too small to make this interaction (Supplementary Figure S6B). The specificity of BcCdnD for cA_3_ formation supports the proposal [24] that the first residue of the ‘XGSX’ motif is the determinant of the major product for the CD-NTases, with asparagine promoting binding of GTP in the acceptor subsite through hydrogen bonding. This is consistent with a mutation of this residue from asparagine to serine, threonine or alanine in EcCdnD where there was a shift in equilibrium towards synthesis of the minor 3ʹ3ʹ3ʹ-cAAA product compared with 3ʹ3ʹ3ʹ-cAAG for the wild type enzyme [24].

The lid residues also provide a clue to substrate specificity and product formation. BcCdnD has a threonine (T150) and a histidine (H155) in the Lid 1 and Lid 2 positions, respectively. The threonine interacts with the base of the nucleotide in the acceptor site and is structurally conserved across the other characterised CdnD enzymes (EcCdnD, SeCdnD, and PaCdnD) and EcCdnC. The Lid 2 residue, however, differs among these enzymes. In EcCdnD and SeCdnD, the residue is a glutamine, which forms either a hydrogen bond with the N3 atom of adenine or two hydrogen bonds with atoms N3 and N2 of guanine in the donor site (Supplementary Figure S6C). BcCdnD, PaCdnD, and EcCdnC all have a histidine in the equivalent position, which is orientated in a way that prevents any interactions with the nucleotide in the donor site (Supplementary Figure S6D). Therefore, for the clade C and D cyclases, those with a glutamine in the Lid 2 position synthesize predominantly 3ʹ3ʹ3ʹ-cAAG, and those with a histidine synthesize 3ʹ3ʹ3ʹ-cAAA. Therefore, there appears to be a key residue responsible for specificity of GTP versus ATP in both the acceptor and donor sites of the cyclases.

Overall, these structures strengthen previous observations on nucleotide binding by the CD-NTase family and expand our understanding of how both ATP and pppApA are accommodated in the acceptor site for the different reaction steps catalysed by the cyclase, as well as the key determinants of substrate specificity in both the donor and acceptor sites.

### Analysis of ATP and pppApA binding by isothermal titration calorimetry

**Figure 3:**
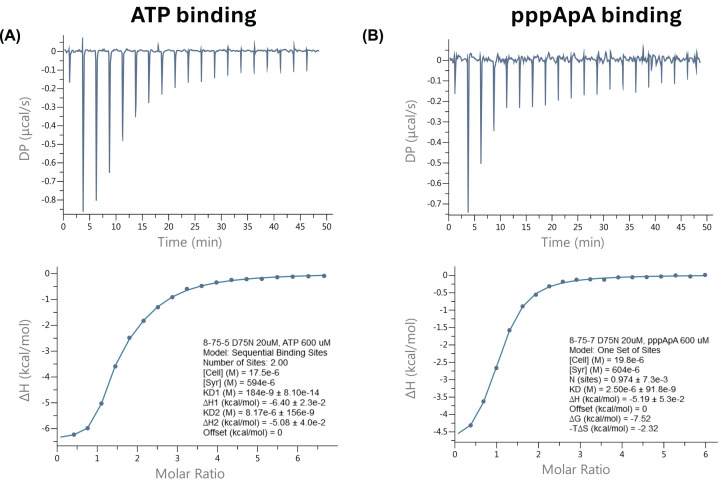
ITC binding isotherms for ATP and pppApA binding to BcCdnD (**A**) Representative ITC thermogram of the BcCdnD D75N variant with a 30-fold higher ATP substrate concentration, fitted to a sequential-binding site model, with two distinct binding sites. Binding sites one and two were found to have respective dissociation constants of 140 ± 40 nM and 5.1 ± 3.2 μM, respectively, across triplicate experiments. (**B**) Representative ITC thermogram of BcCdnD D75N variant with 30-fold higher pppApA concentration, fitted to a one binding site model. Experiments were conducted in duplicate with the number of binding sites determined to be 1.0 ± 0.03 and a dissociation constant of 2.6 ± 0.1 μM.

To quantify the binding of nucleotides to the cyclase, we measured the equilibrium binding affinities for ATP and pppApA to the D75N variant of BcCdnD, which is completely catalytically inactive (Supplementary Figure S2), using isothermal titration calorimetry (ITC) (Figure 3). The binding isotherm for ATP was fitted to a sequential-binding sites model, yielding dissociation constants of 140 ± 40 nM and 5.1 ± 3.2 μM for the high and low affinity sites, likely corresponding to the donor and acceptor sites, respectively. We also investigated binding of pppApA, which binds in the acceptor site (Figure 2). These data fitted best to a single binding site model with a dissociation constant of 2.6 ± 0.1 μM (Figure 3B). The broadly similar dissociation constants observed for ATP and pppApA at the acceptor site are consistent with the conserved interactions observed between these ligands and the protein (Figure 2). Overall, these data point to a very high affinity binding site for ATP in the donor site and a weaker and more generalist acceptor site.

### Kinetic analysis of cA_3_ production reveals substrate inhibition by ATP

**Figure 4:**
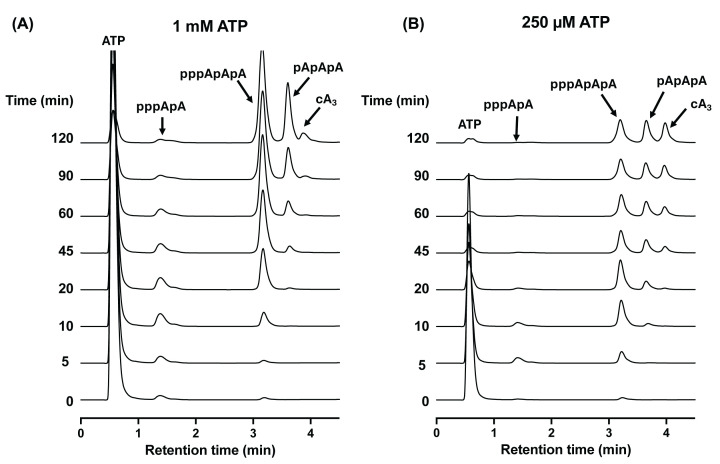
Cyclase activity at (A) 1 mM and (B) 250 μM ATP Reaction products were separated by HPLC, observed by UV spectrometry and identified by retention time with respect to standards, or by MS–MS, as detailed in the ‘Methods’ section. The main product peaks are labelled.

The cyclase reaction was investigated by incubating BcCdnD with two concentrations of ATP (1 mM and 250 μM) for up to 2 h and analysing the products by HPLC (Figure 4). Individual product peaks were identified using standards for pppApA and cA_3_, or by MS–MS in the case of pppApApA and pApApA (Supplementary Figures S7 and S8). At the higher ATP concentration, the final yields of cA_3_ were lower, and the linear trinucleotide intermediates pppApApA and pApApA were observed to build up during the time course of the reaction. pppApApA is the final intermediate before cyclisation to generate cA_3_, while pApApA is presumably a side product of the enzyme. Quantification of the HPLC peaks allowed approximate values for the BcCdnD catalytic rate (*k*_cat_) for cA_3_ synthesis to be estimated as 1 h^−1^ at 250 μM ATP, while pppApApA synthesis had a *k*_cat_ of ∼ 1 min^−1^ at 1 mM ATP.

**Figure 5:**
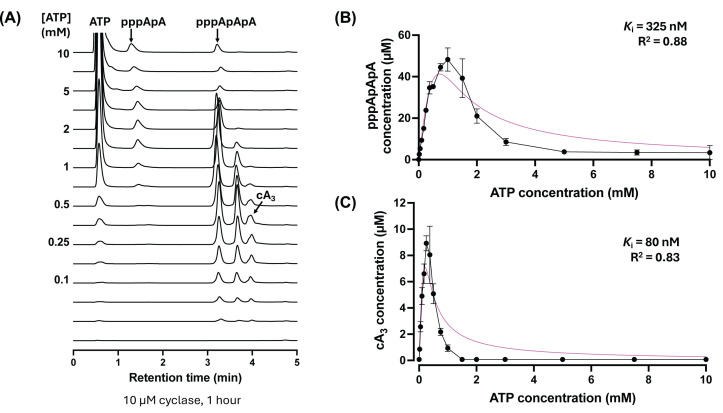
Analysis of substrate inhibition of cyclase activity (**A**) HPLC traces showing reaction products after 1 h reaction with varying ATP starting concentration. (**B**) Quantification of pppApApA production as a function of [ATP], fitted to a substrate inhibition model. (**C**) Quantification of cA_3_ production as a function of [ATP], fitted to a substrate inhibition model. All reactions were carried out in triplicate, and standard deviations are shown.

To investigate ATP inhibition in more detail, we incubated the cyclase with a range of ATP concentrations (20 μM to 10 mM) for 1 h, then analysed the reaction products by HPLC (Figure 5A). Strikingly, at the highest ATP concentrations (5 and 10 mM), no cA_3_ product was observed after the 1 h reaction. Instead, we observed an accumulation of pppApA and, in particular, pppApApA. As the ATP concentration was lowered, more cA_3_ was observed, reaching a maximum at 500 μM ATP. Below this ATP concentration, the cA_3_ product peak began to predominate over the pppApApA intermediate. By quantifying the products as a function of ATP concentration in triplicate experiments, we could fit the data to a standard model for substrate inhibition by ATP, yielding a *K*_i_ of 325 nM for pppApApA synthesis (Figure 5B) and 80 nM for cA_3_ synthesis (Figure 5C). To assess substrate inhibition using an alternative approach, we measured cA_3_ production by activating the relevant CBASS Cap4 effector. cA_3_-activated Cap4 cleaves a model dsDNA molecular beacon, alleviating quenching and resulting in a fluorescent signal (Supplementary Figure S9). Strong ATP inhibition was once again observed, and the data could be fitted to an apparent ATP inhibition constant *K*_i_ of 150 nM, in reasonable agreement with the value obtained using HPLC. Deviations between the fit and the data could reflect secondary inhibition effects by side products such as pApApA and/or non-productive binding of intermediates. This is an interesting area for further analyses.

## Discussion

**Figure 6:**
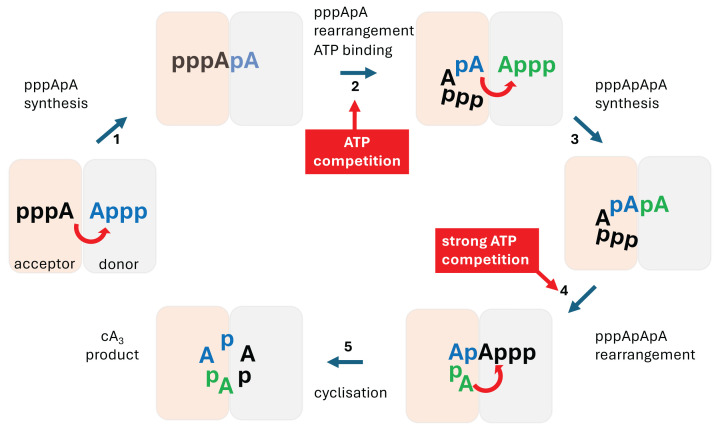
BcCdnD reaction cycle (**1**) Cyclase with ATP bound in the donor and acceptor sites generates pppApA. (**2**) pppApA rearranges in the acceptor site, and ATP binds in the donor site. (**3**) pppApApA formation. (**4**) pppApApA rearranges. (**5**) pppApApA cyclises to form cA_3_. At step 2, ATP can compete with pppApA for binding at the acceptor site. Step 4 is subject to strong competition by ATP binding in the high affinity donor site. The pyrophosphate reaction product is omitted for clarity.

The data presented lead to the following model for the BcCdnD reaction cycle (Figure 6). Initially, ATP binds in the donor and acceptor sites (step 1), leading to the formation of pppApA. This intermediate must rearrange, allowing binding of another ATP molecule in the acceptor site (step 2), leading to pppApApA synthesis (step 3). Step 3 is subject to competition by ATP, which binds at the acceptor site with a similar affinity to pppApA. The modest increase in the pppApA peak at high ATP concentrations (Figure 5A) suggests that the cyclase may at least partly retain pppApA in the active site during rearrangement, reducing competition from ATP. Once formed, linear pppApApA must rearrange and rebind (step 4) in an orientation that supports cyclisation to form the final cA_3_ product (step 5). Step 4 is subject to a high level of substrate inhibition, as ATP will compete with pppApApA for binding, forcing the enzyme back into the reaction cycle for pppApA or pppApApA formation. This model is consistent with the observation of specificity determinants for ATP or GTP in both the donor and acceptor sites, as enzymes that synthesise cAAG must first bind GTP in the acceptor site, while in the final reaction step they need to accommodate the guanine base of pppGpApA in the donor site. If reaction intermediates such as pppApA and pppApApA were retained in the binding site, strong competitive inhibition by ATP would not be so apparent.

These data thus allow a mechanistic understanding for the strong levels of ATP inhibition observed in the BcCdnD cA_3_ synthesis reaction *in vitro*. Given the high (mM) concentration of ATP prevalent in bacterial cells [37,38], cA_3_ synthesis will be effectively shut down under normal conditions. Considering the toxic consequences of cA_3_ activation, this is clearly beneficial in uninfected cells, but the question arises: how does the cyclase overcome ATP inhibition when CBASS defence is activated in late stages of infection? Two possibilities come to mind. The first is simply that depletion of cellular ATP at late stages of phage infection results in de-repression of the cyclase. This is a superficially attractive hypothesis, as it would provide a neat way to sense infection by a broad range of phages. There is ample evidence (recently reviewed in [39]) that some defence systems operate by depleting nucleotides to hinder phage replication [40–42] and cellular ATP levels are known to drop by an order of magnitude or more (to around 100 μM) in *E. coli* cells in late stationary phase [43]. However, the literature on cellular ATP levels during phage infections is sparse and does not come to a consensus, possibly because it has been difficult to quantify ATP concentrations accurately in cells that are infected by phage. Early studies noted drops of 90% in ATP levels in *E. coli* during phage T5 infection [44]. On the other hand, *E. coli* infected with phages P1 or T4 showed no depletion of ATP during the time course of infection [40,45]. ATP liberated from cells on phage-induced lysis has even been used to select active phages for phage therapy [46].

An alternative hypothesis is that conformational changes in the cyclase itself, for example due to conjugation, self-assembly, ligand binding or protein modification, change the geometry of the active site such that turnover number is increased and reaction intermediates are retained, rather than diffusing to bulk solvent. In this situation, ATP would no longer function as a strong competitive inhibitor, and the cyclase activity would be increased significantly. Indeed, BcCdnD function *in vivo* relies on the Cap2/Cap3 conjugation/deconjugation machinery, an aspect that is still poorly understood at a mechanistic level [30]. Active site remodelling is better understood in metazoan cGAS, where dsDNA binding reorganises the active site to promote productive catalysis [47,48]. In the literature, CD-NTases have often been studied using buffers that deviate from physiological conditions, for example, using a pH of 9.4 [11]. These unusual conditions could be mimicking the changes that occur during activation to some extent.

A further point for consideration is how general substrate inhibition might be in the regulation of CBASS CD-NTases? In the case studied here, the formation of a linear trinucleotide that must reorientate in the active site for the final cyclisation step represents an exquisite point of sensitivity for competitive inhibition. In contrast, dinucleotide cyclases may not suffer the same constraints, and it is notable that the *in vitro* activities of those studied are considerably faster than BcCdnD [24]. Nevertheless, the entire CD-NTase family functions by sequential phosphodiester bond formation, where a linear intermediate is formed and must reorientate in the active site prior to the second catalytic step [24], making them all potentially sensitive to competitive inhibition by a nucleotide triphosphate, binding with high affinity in the donor site. This would apply to the nucleotides other than ATP, for cyclases that use them, although their bulk concentrations in cells are comparatively lower. The inhibition of CD-NTases by physiological ATP levels in uninfected cells could thus provide a mechanism to reduce the toxicity of CBASS defence.

## Methods

### Cloning, mutagenesis, and protein expression

Codon-optimised gene sequences encoding BcCdnD and Cap4 were obtained from Integrated DNA Technologies as gBlocks™ and cloned into the expression plasmid pEV5hisTEV [49]. Variant CdnD proteins D73N and D75N were created by site-directed mutagenesis of the expression plasmid. Following sequencing, plasmids were transformed into *E. coli* C43 cells for protein expression. Protein expression was induced with 0.4 mM IPTG, and cultures were incubated with shaking for 4 h at 37°C. Cells were harvested via centrifugation at 4000×***g*** for 15 min and the pellet stored at –20°C until needed.

### Wild-type and variant BcCdnD purification

Cell pellets were thawed and resuspended in lysis buffer (50 mM Tris–HCl, 0.5 M NaCl, 10 mM imidazole, 10% glycerol, pH 7.5) supplemented with 1 mg/ml lysozyme (Sigma–Aldrich) and protease inhibitor cocktail (Roche), and lysed by sonication on ice, as described previously [49]. The crude cell lysate was centrifuged at 120,000×***g*** for 30 min, and the supernatant was loaded onto a Ni^2+^ nitrilotriacetic acid column (His-trap FF crude, Cytiva) pre-equilibrated with wash buffer (50 mM Tris–HCl, 0.5 M NaCl, 10% glycerol, pH 7.5) supplemented with 30 mM imidazole for immobilised metal ion-affinity chromatography. The column was washed with ∼20 column volumes of wash buffer, and target protein was eluted with an imidazole step gradient with elution buffer (50 mM Tris–HCl, 0.5 M NaCl, 0.5 M imidazole, 10% glycerol, pH 7.5) on an ÄKTA Purifier (GE Healthcare Life Sciences). Relevant protein fractions were pooled, and the His-tag was cleaved using TEV protease overnight (10:1 w/w ratio) during dialysis (Biodesign Cellulose Dialysis Tubing Roll, 10 kDa) against wash buffer (100:1 w/w ratio). The cleaved protein was reapplied to a fresh HisTrap FF column and eluted in wash buffer (‘unbound fraction’). The protein was pooled, concentrate, and applied to HiLoad Superdex 200 pg column (16/60 or 26/60) pre-equilibrated with gel filtration buffer (20 mM Tris–HCl, 250 mM NaCl, 10% glycerol, pH 7.5) for size exclusion chromatography. Protein-containing fractions were analysed on SDS–PAGE before combining and concentrating to ∼10 mg/ml, aliquoted, and flash-frozen in liquid nitrogen for storage at −70°C. Purified proteins are shown in Supplementary Figure S1.

### Cyclase cOA production assay

Fifty microliters reactions of 10 μM BcCdnD were incubated with 250 μM ATP for 1 h at 37°C, in cyclase assay buffer (20 mM HEPES, pH 7.5, 150 mM NaCl, 10 mM MgCl_2_) unless otherwise stated. BcCdnD reactions were quenched with 2 volumes of CHROMASOLVTM HPLC-grade methanol (Honeywell Riedel-de Haën™) and vortexed for 30 s to precipitate protein. Protein precipitate was removed by centrifugation for 10 min at 10,000×***g***. The solvent from the supernatant fraction was evaporated in a Concentrator Plus (Eppendorf) at 30°C and nucleotides resuspended in UHPLC grade water (Fisher Scientific) for analysis by HPLC or fluorescent endonuclease assay.

### HPLC

Five microliters of BcCdnD product or synthetic standard was injected onto a C18 column (Kinetex® 2.6 μm EVO C18 100 Å, LC column 50 × 2.1 mm) attached to a Thermo UltiMate 3000 chromatography system. Absorbance was monitored at 260 nm and 40°C. Gradient elution was performed with solvent A (100 mM ammonium acetate) and solvent B (methanol + 0.1% trifluoroacetic acid) with a flow rate of 0.3 ml min^−1^ as follows: 0–0.5 min, 0% B; 0.5–3.5 min, 20% B; 3.5–5 min, 50% B; 5–10 min, 100% B. The area of the peak (mAU (milli-absorbance units) × min) was determined with the Chromeleon 6.80 software (Dionex). Standard curves were generated for cA_3_ and ATP and used to calculate nucleotide concentrations of remaining ATP, cA_3_ produced, and pppApApA intermediate (using cA_3_ standard curve) by fitting integrated peak areas. Concentrations were normalised to an internal cA_6_ standard, and SDS–PAGE band intensity was calculated using Fiji (https://fiji.sc) to account for differences in HPLC injection volume and BcCdnD concentration between variants, respectively. Data were analysed and visualised using GraphPad Prism 9.5.1 (Dotmatics).

### LC-MS/MS

Liquid chromatography tandem mass spectrometry (LC-MS/MS) was performed on an Eksigent 400 LC system coupled to a Sciex 6600 QTOF mass spectrometer, operated in trap-elute mode at microflow rates. Samples were injected into a YMC Triart C18 trap cartridge (0.5 × 5.0 mm) using 99.95% H_2_O with 0.05% TFA at a flow rate of 10 μl/min for 3 min to remove salts (diverted to waste). The trap was subsequently brought in-line with the analytical column (YMC Triart, 150 × 0.075 mm). Chromatographic separation was achieved using a gradient elution with solvent A (99.9% H_2_O with 0.1% formic acid) and solvent B (80% acetonitrile, 20% H_2_O and 0.1% formic acid) at a flow rate of 5 μl/min, following 0 min, 3% B; 0–6 min, 3-95% B; 6–8 min, hold at 95% B; 8–9 min, return to 3% B; 9–13 min, re-equilibrate at 3% B. The elution was directly introduced into the ESI turbospray of the mass spectrometer. Data were acquired in positive ion mode over a mass range of m/z 120–1000. Selected precursor ions were subjected to CID fragmentation using collision energies between 25 and 45 V, and product ion spectra were collected.

### Cap4 nuclease assay

Methods and conditions were adapted from the previously described NucC assay [50]. Briefly, Cap4 nuclease assays were conducted in a FluoStar Omega plate reader (BMG Labtech) using fluorescence detection (excitation/emission wavelength 485/520 nm) in black non-binding half-area 96-well plates (Corning). Fluorescence was measured at 30 s intervals at 37°C. A 40× fold dilution of extracted BcCdnD product or cA_3_ synthetic standard was pre-incubated with 100 nM 30 bp dsDNA substrate where one strand has a FAM fluorophore and the other an IowaBlack quencher in nuclease buffer (50 mM Tris–HCl, 20 mM NaCl, 10 mM MgCl_2_, 10% (v/v) glycerol, 10% (v/v) PEG 8000, pH 8) for 10 min at 37°C to generate the initial baseline. Following incubation, measurement was paused, and 500 nM Cap4 nuclease was added before measurements were continued for another 50 min.

### ITC

The dissociation constants and binding stoichiometry of cyclase binding events with ATP and pppApA were determined by ITC using a MicroCal PEAQ-ITC calorimeter (Malvern Panalytical). The catalytically inactive D75N BcCdnD variant was used for ITC experiments. BcCdnD D75N protein was dialysed in 2 kDa MWCO Slide-a-Lyzer^™^ mini-dialysis units (Thermo Fisher Scientific) overnight against ITC buffer (HEPES, pH 8.0, 150 mM KCl, 10 mM MgCl_2_) prior to dilution in dialysate to 20 μM. ATP was resuspended and diluted to 600 μM in dialysate, while 10 mM pppApA (in water) was diluted to 600 μM in dialysate. ITC titration was conducted at 25°C, with an initial 0.4 μl injection of nucleotide, followed by 18 subsequent 2 μl nucleotide injections, with stirring at 750 rpm and 150 s between injections. MicroCal PEAQ-ITC analysis software (Malvern Panalytical) was used for baseline correction, integration, and fitting of pppApA curves to single-binding site model and ATP curves to sequential binding with two sites model. All ITC experiments were carried out in duplicate or triplicate, and representative isotherms including alternative binding models are shown (Supplementary Figure S10).

### Crystallisation

Initial crystallisation conditions for wild-type BcCdnD with ATP and BcCdnD D73N variant with pppApA (BIOLOG Life Sciences Institute) and pppApA + ATP were obtained from sparse matrix screening of the protein–ligand mixtures on a nano-litre scale. A molar excess of ATP, pppApA, or pppApA + ATP was added to a solution of 10 mg/ml BcCdnD and incubated at 277 K for 30 min prior to centrifugation at 20 000×***g***, 277 K, in preparation for crystallisation trials. The protein–ligand solution was mixed in 1:1 and 2:1 protein-to-mother liquor ratios in sitting drop plates. The plates were sealed and left to equilibrate by vapour diffusion at 293 K. Diffraction-quality crystals grew in a variety of conditions, each of which was cryoprotected in mother liquor plus 25% glycerol prior to cryo-cooling in liquid nitrogen in preparation for data collection. The best crystals for BcCdnD incubated with ATP originated from a condition containing 1.6 M ammonium sulphate, 0.1 M Bis-Tris (pH 5.5); BcCdnD with pppApA from 20% PEG 3000, 0.1 M sodium citrate (pH 5.5); and BcCdnD with ATP + pppApA from 2.4 M sodium malonate dibasic monohydrate (pH 7).

### X-ray data collection and processing

X-ray data were collected at Diamond Light Source, Oxfordshire, on beamlines I03 and I04, at a wavelength of 0.97623 or 0.9537 Å, at 100 K. All data were automatically processed using Xia2 [51] with DIALS [52] or AUTOPROC [53] employing the STARANISO [54] strategy to 2.12 Å resolution (BcCdnD WT with ATP), 2.83 Å resolution (BcCdnD D73N with pppApA) and 2.09 Å resolution (BcCdnD D73N with ATP + pppApA). Data were phased using PhaserMR [55] in the CCP4 suite [56] utilising a model generated by AlphaFold2 [57], with initial B-factors modelled in Phenix [58]. The three models were refined in the same manner, using iterative cycles of REFMAC5 [59] or phenix.refine [58] with manual model manipulation in COOT [60]. For each ligand-bound structure, electron density was clearly visible in the maximum likelihood/σA-weighted *F*_obs_–*F*_calc_ electron density map at 3σ. Coordinates for pppApA were generated in ChemDraw (Perkin Elmer), and the library was generated using acedrg [61] before fitting of the molecule in COOT. Model quality was monitored throughout using Molprobity [62]. Structural comparisons were calculated using PDBeFold [63]. Ramachandran and refinement statistics for each structure are presented in Supplementary Table 1. The coordinates and data have been validated and deposited in the Protein Data Bank with deposition codes 9SA4, 9SA5, and 9SAX.

## Supplementary Material

Supplementary Figures S1-S10 and Supplementary Table S1

Supplementary Materials

## Data Availability

The protein structure coordinates and data have been deposited in the Protein Data Bank with deposition codes 9SA4, 9SAX, and 9SA5 [64]. Underlying data for Figures 4 and 5 are provided as a supplementary data file.

## Open Access

Open access for this article was enabled by the participation of the University of St Andrews in an all-inclusive Read & Publish agreement with Portland Press and the Biochemical Society under an agreement with JISC.

## Abbreviations

BcCdnD: *Bacillus cereus* CD-NTase
CBASS: cyclic oligonucleotide based anti-phage signalling system
CD-NTase: cGAS/DncV-like nucleotidyltransferase
cNT: cyclic nucleotide
DncV: dinucleotide cyclase in *Vibrio*
ITC: isothermal titration calorimetry
LC-MS/MS: liquid chromatography tandem mass spectrometry
mAU: milli-absorbance units
RMSD: root mean square deviation
SMODS: second messenger oligonucleotide or dinucleotide synthetase
