# Supplementary material for: Inhibition by ATP regulates the activity of a CBASS anti-phage nucleotide cyclase
Source: Biochem J. 2026 Jul 30;483(8):1617–30. doi: 10.1042/BCJ20260457 (PMC13424991; doi:10.1042/BCJ20260457)
Supplement: Supplementary Figures S1-S10 and Supplementary Table S1 [file BCJ-2026-0457_supp.pdf]

Inhibition by ATP regulates the activity of a CBASS anti-phage nucleotide cyclase

Laura Gaskell-Mew, Stuart McQuarrie, Stephen A. McMahon, Peter Wotherspoon, Shirley Graham, Tracey M Gloster and Malcolm F White**

	ATP	pppApA	ATP + pppApA
**Data processing**			
Space group	$P2_12_12_1$	$P2_12_12_1$	$P2_12_12_1$
Cell dimensions			
a, b, c (Å)	86.8 115.6 156.5	83.9 117.0 155.9	87.3 116.7 156.6
α, β, γ (°)	90 90 90	90 90 90	90 90 90
Resolution (Å)	100.0 – 2.12 (2.15 – 2.12)*	73.89 – 2.83 (3.12 – 2.83)*	78.34 – 2.09 (2.12 – 2.09)*
R_{merge}	0.12 (3.28)*	0.11 (1.15)*	0.10 (1.24)*
$I/σ(I)$	9.3 (0.2)*	9.8 (1.4)*	10.1 (0.3)*
Completeness (%)	100 (97.7)*	66.1 (13.2)* spherical 91.1 (60.7)*ellipsoidal	96.0 (71.0)*
Average redundancy	13.6 (14.0)*	6.5 (6.4)*	5.6 (2.4)*
$CC_{1/2}$	0.99 (0.32)*	0.99 (0.56)*	0.99 (0.19)*
V_m ($Å^3$/Da)	2.60	2.54	2.65
Solvent (%)	52.8	51.6	53.6
**Refinement**			
Unique reflections	90060 (4369)	24687 (1234)	91646 (3347)
R_{work} / R_{free}	20.4 / 25.6	21.7 / 26.2	21.8 / 27.7
Geometric deviations			
Bonds (Å) / Angles (°)	0.008 / 0.98	0.006 / 1.33	0.009 / 1.009
No. atoms (non H)			
Protein	9681	9219	9754
Water	212	21	235
ATP	248	N/A	93
pppApA	N/A	212	191
Mg	4	N/A	6
SO_4	55	N/A	N/A
PO_4	N/A	20	N/A
B factors ($Å^2$)			
Protein	68.0	88.1	52.8
Water	59.1	53.3	47.3
ATP	85.4	N/A	73.9
pppApA	N/A	118.4	83.8
Mg	70.3	N/A	61.8
SO_4	86.8	N/A	N/A
PO_4	N/A	111.2	N/A
Ramachandran			
Favoured / outlier (%)	98.9 / 0	96.4 / 0	98.4 / 0
Molprobity score / centile (%)	1.09 / 100	1.29 / 100	1.14 / 99
PDB Code	9SA4	9SAX	9SA5

* Values in parentheses are for the highest-resolution shell.

Supplementary Table 1. Data processing and refinement statistics for BcCdnD in complex with ATP, pppApA or ATP + pppApA.

Inhibition by ATP regulates the activity of a CBASS anti-phage nucleotide cyclase

Laura Gaskell-Mew, Stuart McQuarrie, Stephen A. McMahon, Peter Wotherspoon, Shirley Graham, Tracey M Gloster and Malcolm F White**

Supplementary Figures

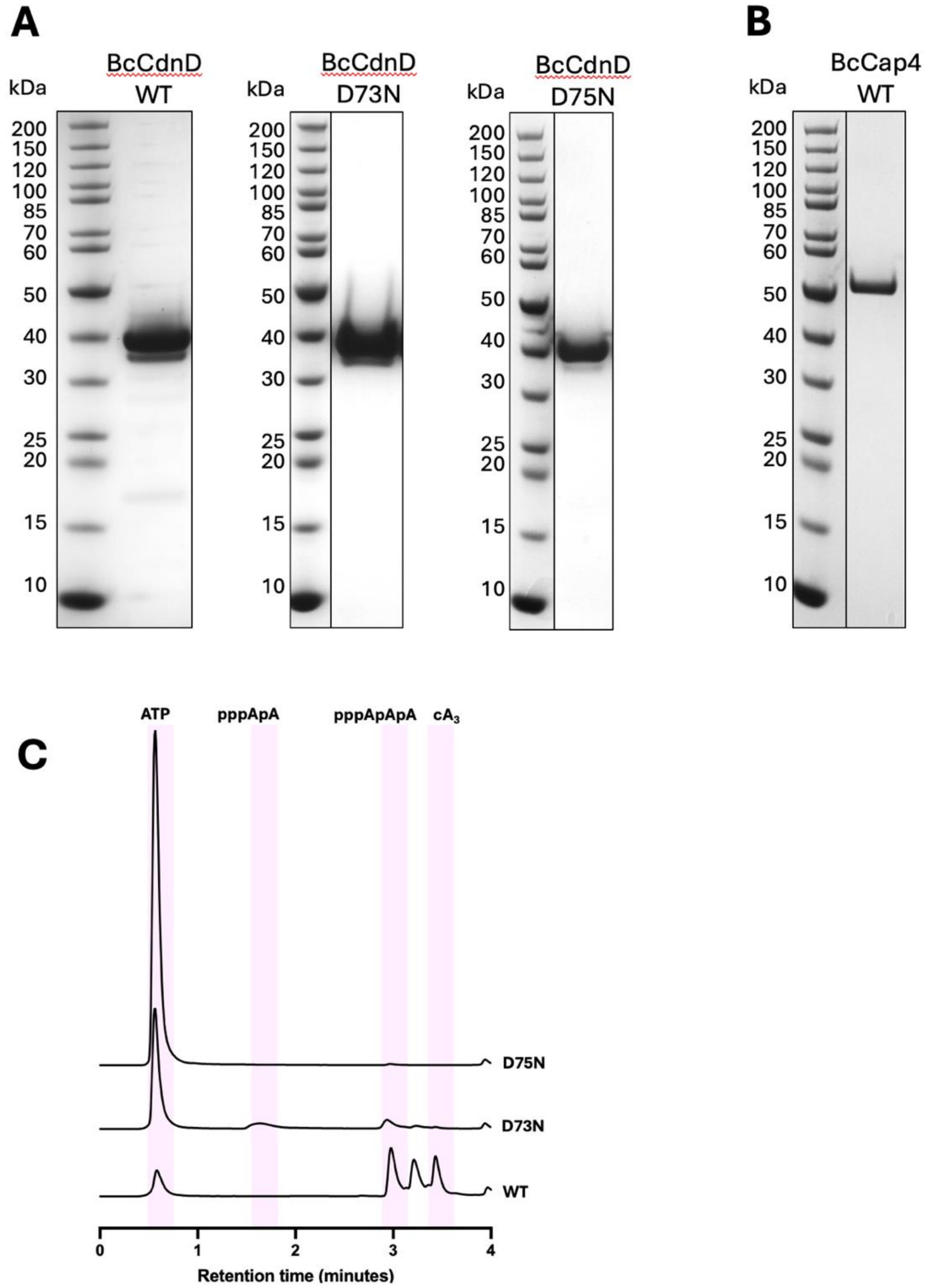

Supplementary Figure 1. SDS-PAGE analysis of purified (A) BcCdnD and (B) Cap4 proteins. Proteins were purified through Ni-IMAC affinity and size exclusion chromatography before running on SDS-PAGE for visualisation. **A.** BcCdnD WT and variants D73N and D75N. **B.** Cap4 WT.**C.** HPLC analysis of BcCdnD WT and variant activity in standard reaction conditions, 250 µM ATP, 60 min. Trace amounts of reaction intermediates were visible for the D73N variant.

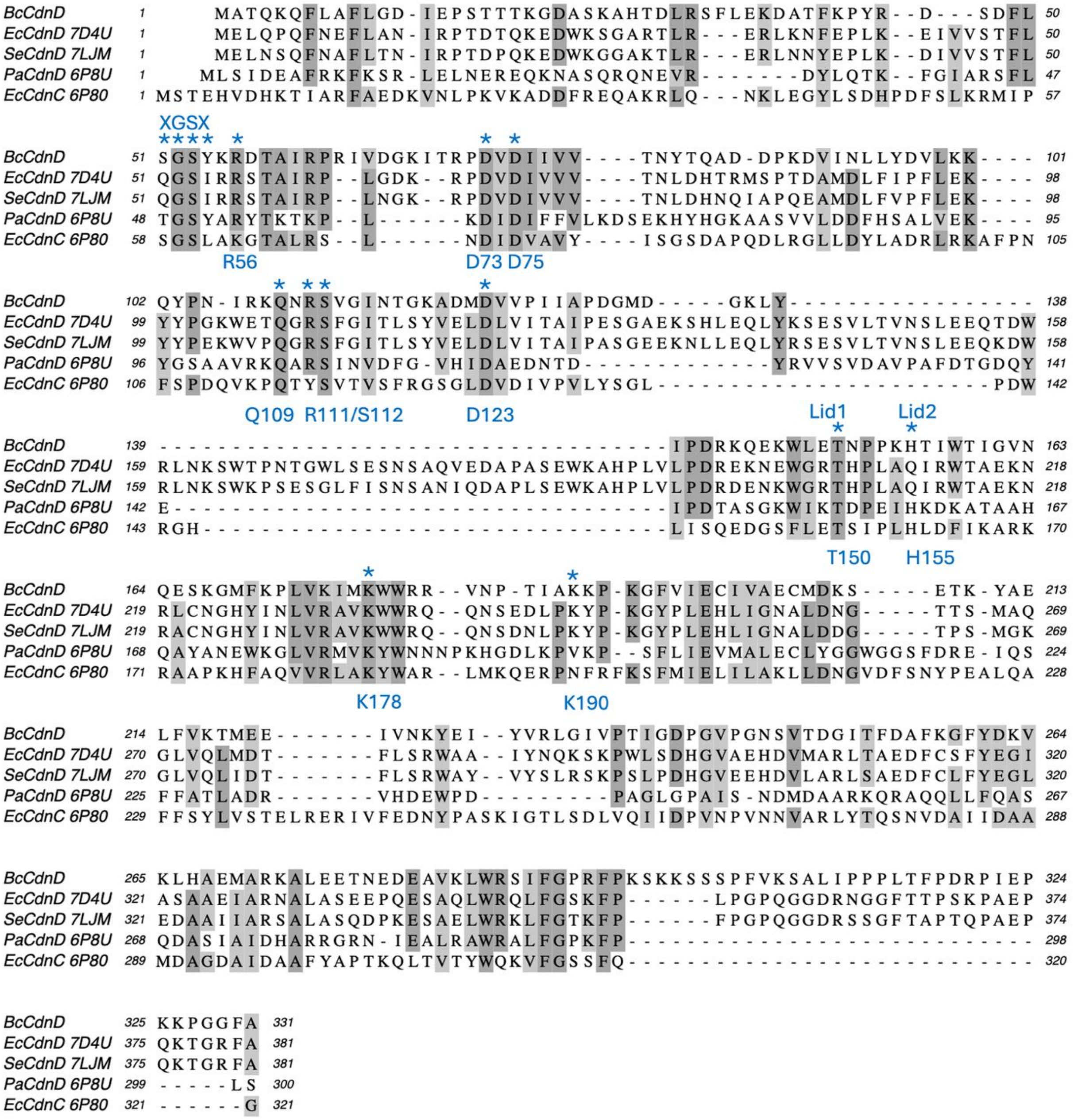

Supplementary Figure 2. Multiple sequence alignment of selected CD-NTases. Residues discussed in the text are labelled. BcCdnD, CdnD from *Bacillus cereus*; EcCdnD, CdnD from *Enterobacter cloacae*; SeCdnD, CdnD from *Salmonella enterica*; PaCdnD, CdnD from *Pseudomonas aeruginosa*; EcCdnC, CdnC from *Escherichia coli*.

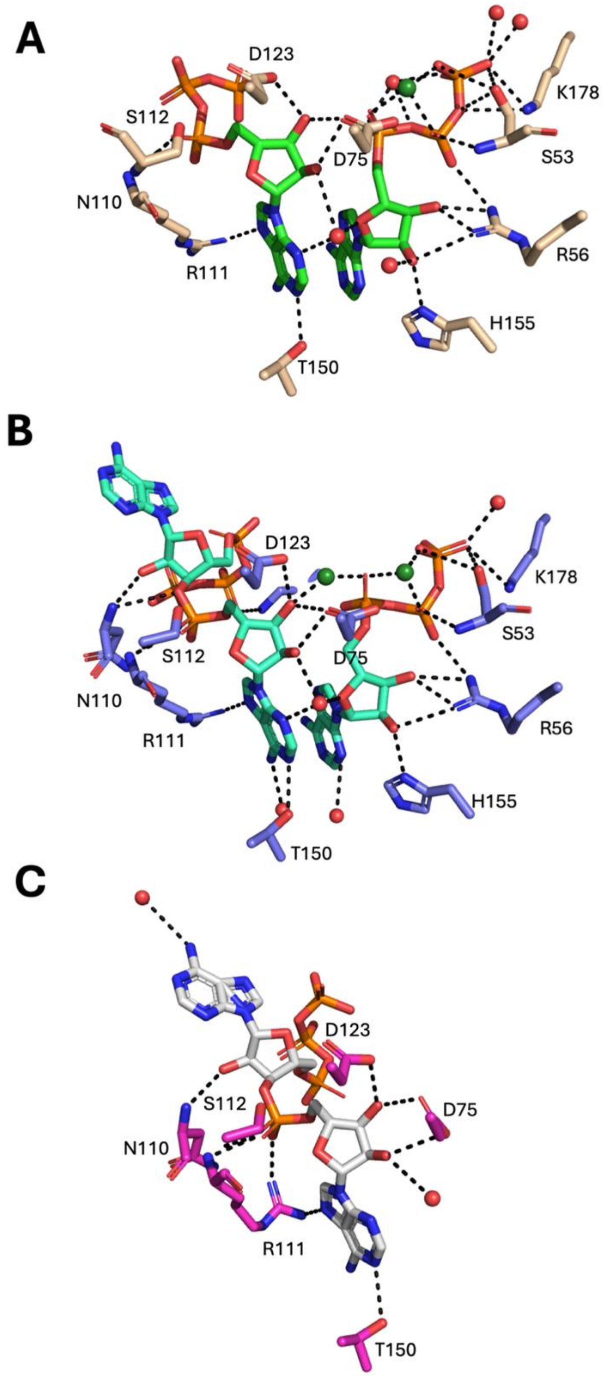

Supplementary Figure 3. Interactions between ligands, metal ions, water molecules, and protein residues for BcCdnD complexes. A. BcCdnD in complex with two ATP molecules. BcCdnD residues are shown in wheat-coloured sticks and ATP molecules in green sticks. **B.** BcCdnD in complex with ATP + pppApA molecules. BcCdnD residues are shown in light blue sticks and ATP molecules in cyan sticks. **C.** BcCdnD in complex with pppApA. BcCdnD residues are shown in magenta sticks and pppApA in grey sticks. In each image the hydrogen bond network formed by the ligands is shown in dashed black lines. Magnesium ions are shown as dark green spheres and water molecules as red spheres. The same orientation has been used for each panel; the acceptor site is on the left and donor site on the right. Main chain atoms have been shown where involved in interactions with the ligands, but otherwise omitted for clarity.

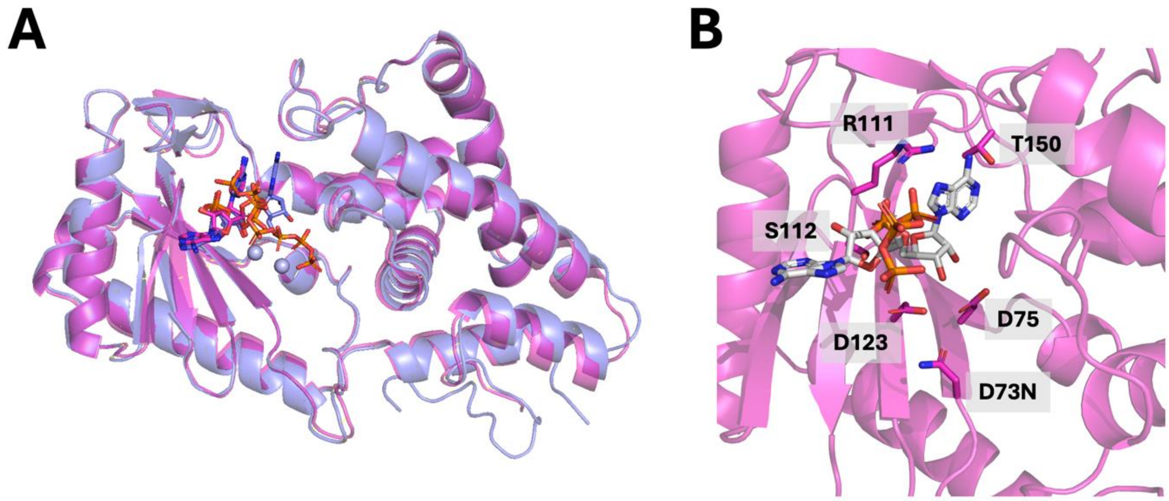

Supplementary Figure 4. Crystal structure of BcCdnD in complex with pppApA. A. Superposition of BcCdnD D73N variant in complex with pppApA in the acceptor site (magenta) and BcCdnD D73N variant in complex with ATP in the donor site and pppApA in the acceptor site (light blue) (RMSD of 0.7 Å over 296 Cα atoms). Ligands and magnesium ions are shown in the same colouring as the protein backbone. **B.** Structure of BcCdnD (D73N variant) co-crystallised with pppApA in the acceptor site. The protein is shown in cartoon (magenta). Conserved residues implicated in binding and catalysis are shown as sticks and labelled. pppApA is shown as sticks (grey).

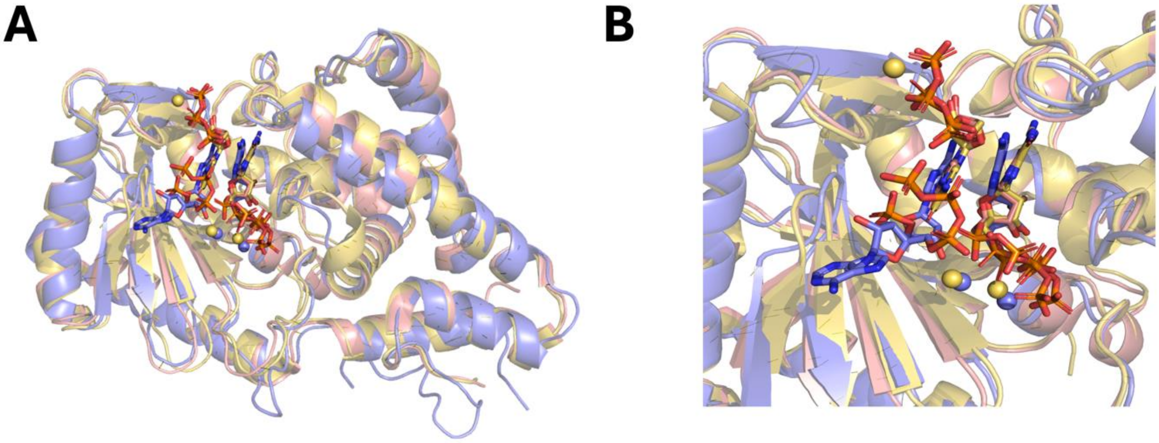

Supplementary Figure 5. Structural alignment of BcCdnD with its closest homologues. A. Superposition of BcCdnD D73N variant in complex with ATP in donor site + pppApA in acceptor site (light blue), CdnD from *Enterobacter cloacae* in complex with ATP in both donor and acceptor sites (pink; PDB 7D4U; RMSD of 1.7 Å over 281 $C\alpha$ atoms) and CdnD from *Salmonella enterica* in complex with GTP in both donor and acceptor sites (gold; PDB 7LJM; RMSD of 1.7 Å over 286 $C\alpha$ atoms). The protein main chain is shown in cartoon, the ligands as sticks, and magnesium ions as spheres. Ligands and magnesium ions are shown in the same colouring as the protein backbone. For orientation, the donor site is on the right and acceptor site on the left. **B.** Closer view of ligands bound in CdnD active site, with all details identical to **A**.

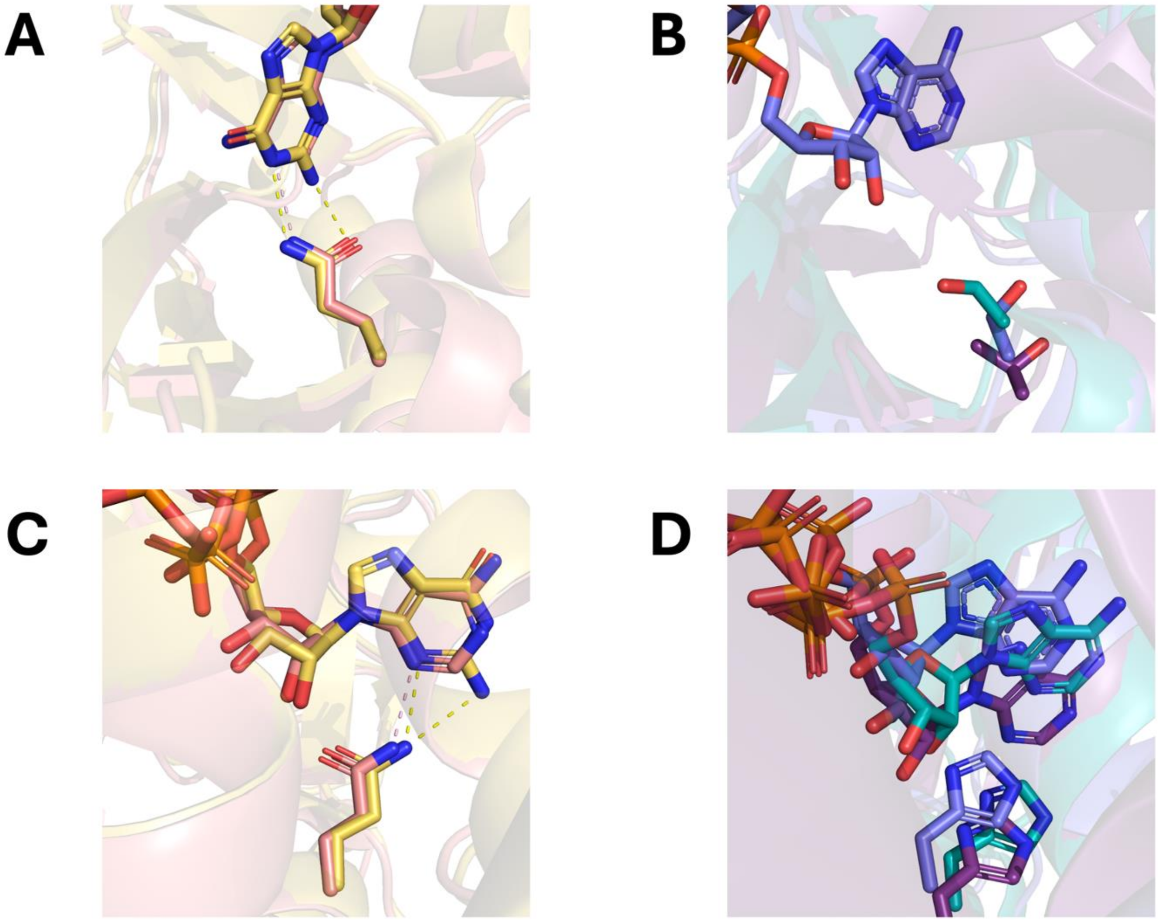

Supplementary Figure 6. Structural alignment of key residues in BcCdnD with its closest homologues. A. Superposition of EcCdnD in complex with ATP in both donor and acceptor sites (pink; PDB 7D4U) and SeCdnD complex with GTP in both donor and acceptor sites (gold; PDB 7LJM). The ligands and conserved glutamine representing the first X residue of the 'XGSX' motif are shown in sticks in the respective colours. The two hydrogen bonds between Q51 in SeCdnD and guanine are shown in gold and the one hydrogen bond between Q51 in EcCdnD and adenine are shown in pink. **B.** Superposition of BcCdnD in complex with ATP in donor site + pppApA in acceptor site (light blue), PaCdnD D62/D64 variant in complex with ATP in donor site (purple; PDB 6P8J) and EcCdnC in complex with ATP (teal; PDB 6P80). The ligands and conserved serine (BcCdnD and EcCdnC) or threonine (PaCdnD) representing the first X residue of the 'XGSX' motif are shown in sticks in the respective colours. Only the acceptor site ligands are shown; ligands in the donor site and metal ions have not been included for clarity. **C.** Superposition of EcCdnD in complex with ATP in both donor and acceptor sites (pink; PDB 7D4U) and SeCdnD complex with GTP in both donor and acceptor sites (gold; PDB 7LJM). The ligands and conserved glutamine representing the Lid 2 residue are shown in sticks in the respective colours. The two hydrogen bonds between Q210 in SeCdnD and guanine are shown in gold and the one hydrogen bond between Q210 in EcCdnD and adenine are shown in pink. **D.** Superposition of BcCdnD in complex with ATP in donor site + pppApA in acceptor site (light blue), PaCdnD D62/D64 variant in complex with ATP in donor site (purple; PDB 6P8J) and EcCdnC in complex with ATP (teal; PDB 6P80). The ligands and conserved histidine representing the Lid 2 residue are shown in sticks in the respective colours. Only the donor site ligands are shown; ligands in the acceptor site and metal ions have not been included for clarity.

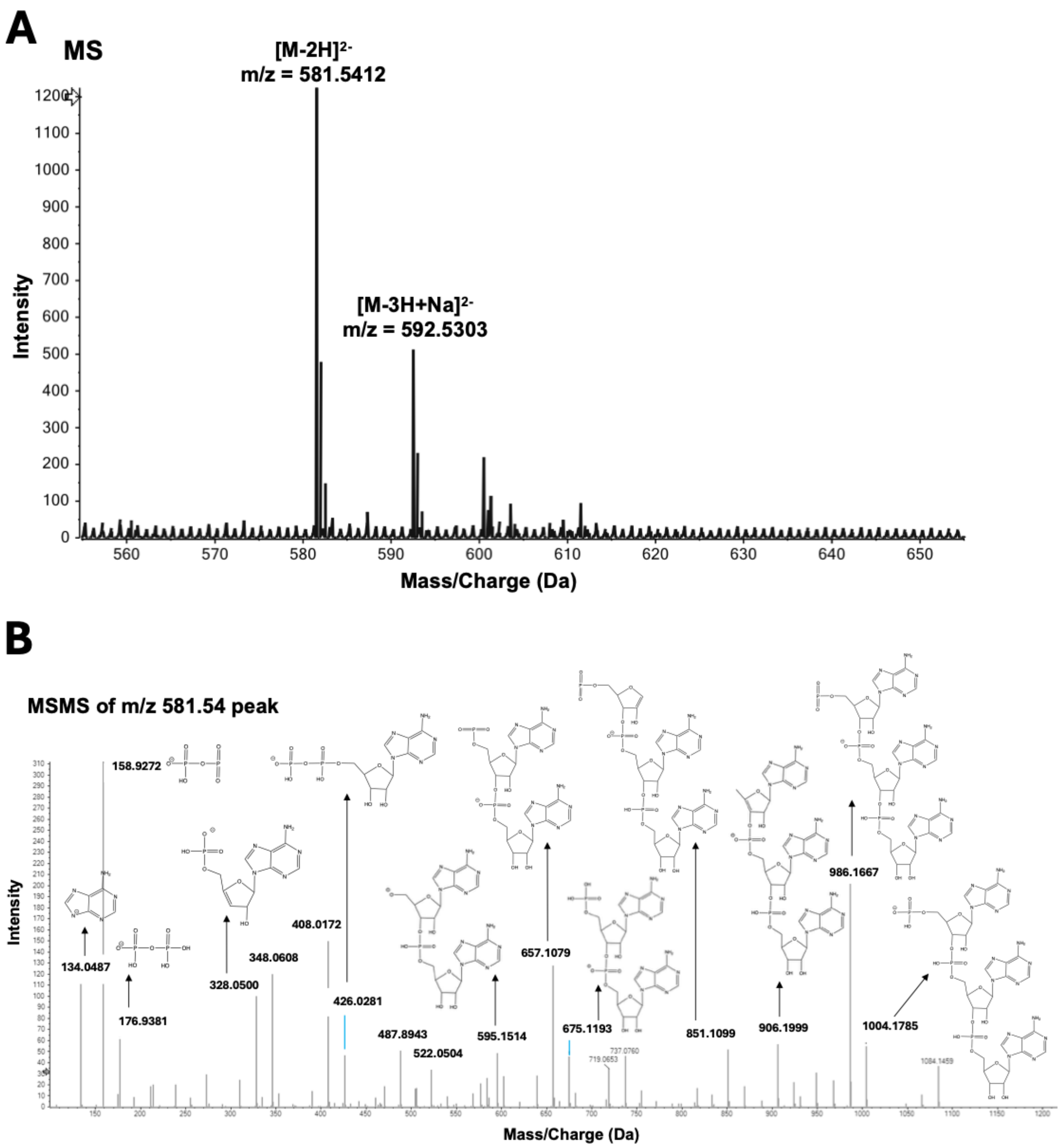

Supplementary Figure 7. Cyclase reaction product pppApApA characterisation by MS and MS-MS.

A. MS spectra of intermediate product with identifying peak with m/z value of 581.5412, with 3.6 ppm difference to predicted pppApApA m/z 581.5434 for doubly charged ion. **B.** MS/MS fragmentation analysis of intermediate product with m/z value of 581.5412. Identified peaks in bold with subset labelled with pppApApA fragment chemical structures.

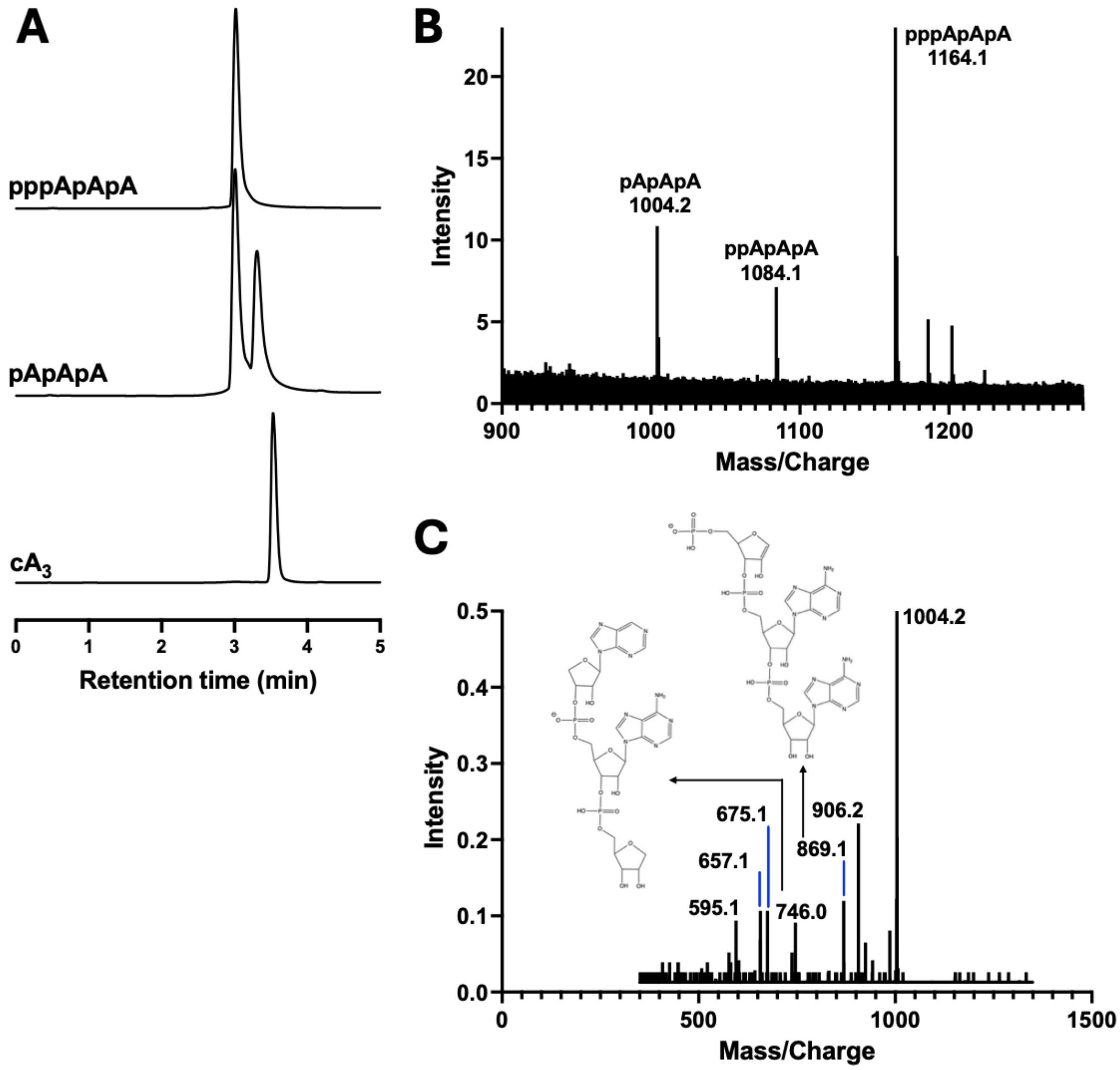

Supplementary Figure 8. Cyclase reaction product pApApA purification and MS analysis.

A. HPLC traces of BcCdnD intermediate pppApApA and synthetic cA_3, alongside product mixture containing pppApApA and potential pApApA displaying retention time between that of cA_3 and pppApApA. **B.** MS spectra of intermediate product with identifying peak with m/z value of 1004.2, for singly charged ion. **B.** MS/MS fragmentation analysis of intermediate product with m/z value of 1004.2. Identified peaks of 746.0 and 869.1 are labelled with pApApA fragment chemical structures, whilst all other labelled peaks displayed bold were previously identified in pppApApA MS/MS fragmentation.

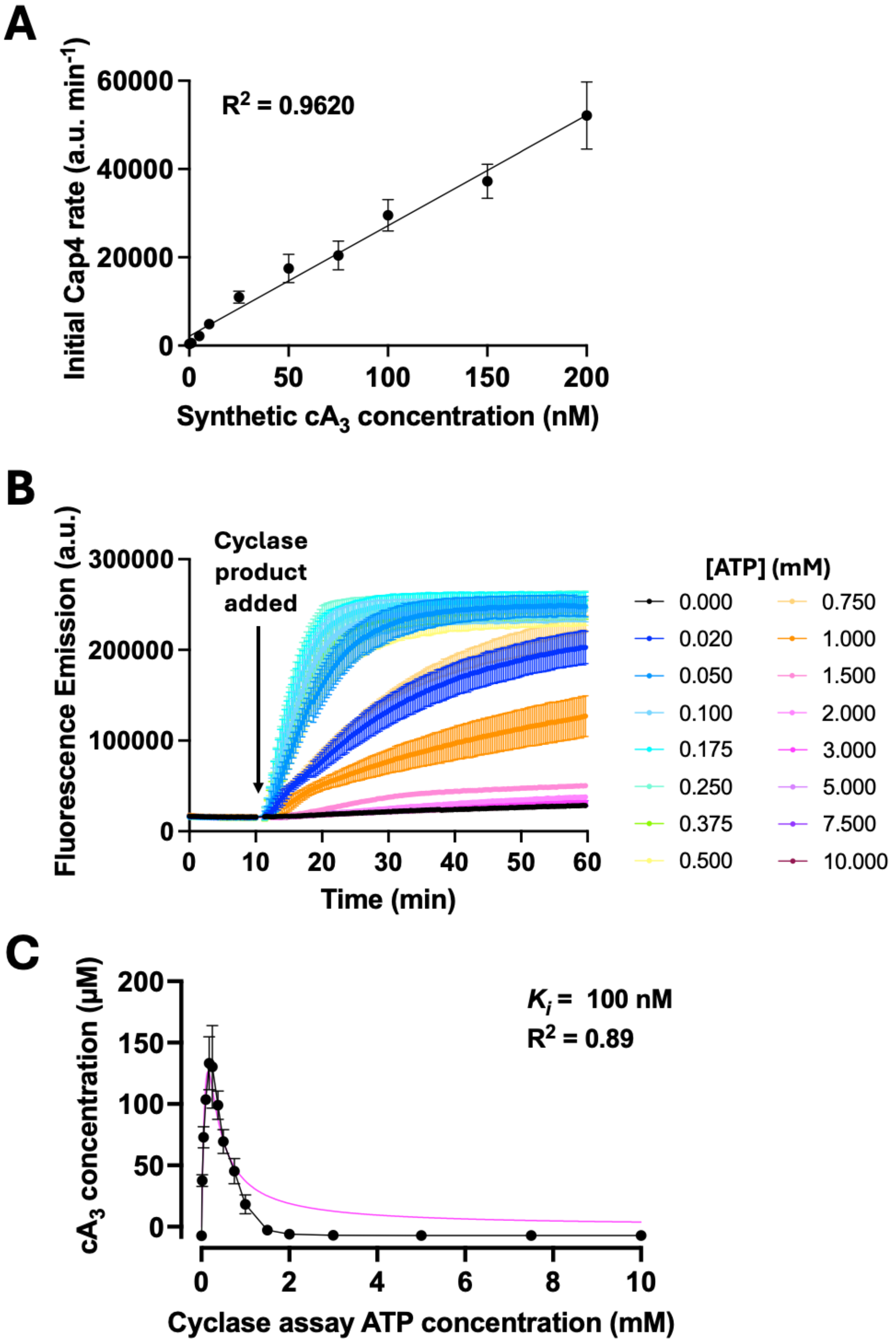

Supplementary Figure 9. Estimation of ATP substrate inhibition using an assay coupled to Cap4 activity. A. *Bce* Cap4 activated by varying concentrations of synthetic cA_3 to generate a standard curve of initial rate of cleavage (a.u. min^{-1}) as a function of [cA_3]. Data were fitted to a linear regression with an R^2 of 0.96. **B.** Cap4 activated with a dilution of BcCdnD product following 1 h incubation with varying ATP starting concentrations. **C.** cA_3 production estimates were generated from initial rates (**B**) and Cap4 standard curve (**A**) and quantified as a function of [ATP], fitted to a substrate inhibition model. All reactions were carried out in triplicate, and standard deviations are shown.
